# Low‐Loss Routing of Strongly Confined Light in Ultra‐Compact Silicon Slot‐Waveguide Bends

**DOI:** 10.1002/nap2.70260

**Published:** 2026-08-20

**Authors:** Ji‐Hwan Park, Jae‐Yong Kim, Seokjin Hong, Berkay Neseli, Hyo‐Hoon Park, Hamza Kurt

**Affiliations:** ^1^ School of Electrical Engineering Korea Advanced Institute of Science and Technology Daejeon Republic of Korea; ^2^ Command, Control, Communications, Computers, Intelligence, Surveillance and Reconnaissance (C4ISR) Systems Center Defense Agency for Technology and Quality (DTaQ) Daegu Republic of Korea

## Abstract

Photonic slot waveguides confine light in subwavelength low‐index gaps and are useful for compact light–matter interaction devices, but tight bending strongly distorts the slot‐confined mode and limits dense on‐chip routing. Conventional routing strategies typically avoid strong curvature by using large bend radii or strip–slot conversion, thereby increasing the circuit footprint. Here, we demonstrate an ultra‐compact slot waveguide bend in which independent rail‐width optimization gives rise to asymmetric mode evolution within the bending region, enabling efficient output‐mode recovery under extreme curvature. The optimized geometry develops a rail‐width asymmetry, leading to field redistribution toward the wider outer rail followed by efficient coupling to the output slot mode. Implemented on a 220‐nm‐thick silicon‐on‐insulator platform, the inverse‐designed 90° bend has a bending radius of 1.5 μm and a 100 nm slot gap. Three‐dimensional finite‐difference time‐domain simulations predict quasi‐TE_0_ modal transmission exceeding 95% across the C‐band. Experimental measurements further confirm broadband transmission in routing structures containing up to 80 cascaded ultra‐tight 90° bends. From the accumulated routing loss, we extract an effective excess loss of approximately 0.29 dB per 90° turn. These results demonstrate asymmetric mode evolution as a practical design strategy for ultra‐compact slot‐waveguide routing in high‐density photonic integrated circuits.

## Graphical abstract

An ultra‐compact asymmetric silicon slot‐waveguide bend enables low‐loss routing of strongly confined light by compensating for curvature‐induced field displacement. The optimized rail‐width profile redistributes the optical field toward the outer rail and restores the slot‐confined mode at the output, achieving broadband quasi‐TE_0_ transmission in a 1.5 μm‐radius bend.

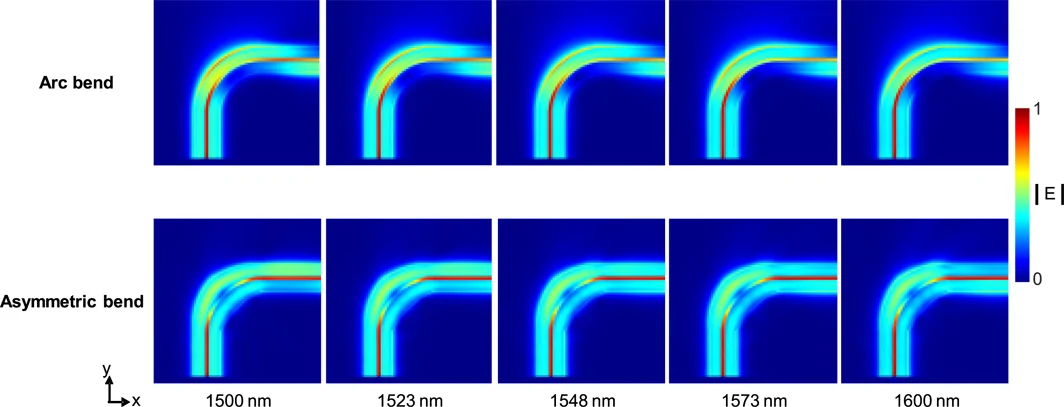

## 1 Introduction

The rapid expansion of photonic integrated circuits (PICs) has intensified the demand for compact, high‐efficiency passive components to enable scalable and high‐density on‐chip architectures [1, 2]. Among essential building blocks such as waveguide bends [3, 4, 5, 6, 7, 8, 9, 10, 11, 12, 13, 14], power splitters [15, 16], mode‐division multiplexing (MDM) devices [17], and resonators [18], increasing attention has been directed toward waveguide platforms that can provide strong optical confinement within a minimal footprint. In this context, dielectric slot waveguides have attracted considerable interest due to their ability to tightly confine light within subwavelength low‐index gaps formed between high‐index rails [19]. They offer a low‐loss and CMOS‐compatible alternative to plasmonic slot waveguides, which suffer from significant ohmic and propagation losses as well as limited compatibility with standard fabrication processes [20]. These unique characteristics enable enhanced light–matter interactions, making dielectric slot waveguides promising candidates for various applications including biosensing [21, 22, 23, 24], near‐field optical probes [25], nonlinear optics [26, 27, 28, 29], and emerging quantum photonic systems [30, 31].

Despite these advantages, slot waveguides remain highly vulnerable to bend‐induced loss under tight curvature. In high‐index‐contrast platforms such as silicon‐on‐insulator (SOI), a small bend radius can strongly perturb the slot‐confined mode, causing modal distortion, outward field displacement, and increased radiation loss [32]. This sensitivity is particularly problematic for dense routing, because the optical field in a slot waveguide is concentrated in a narrow low‐index gap and is therefore easily distorted when the local propagation direction changes rapidly. Analytical and semi‐analytical studies have provided useful insight into the modal properties of bent slot waveguides [33, 34, 35], but practical low‐loss routing at ultra‐small radii remains challenging. Existing implementations therefore often avoid highly curved slot sections by using relatively large bend radii or by transferring light between slot and strip waveguides through strip–slot mode converters [36, 37, 38, 39]. Although effective, these approaches increase the footprint and structural complexity of slot‐based photonic circuits and generally do not exploit the additional design freedom available within the slot‐bend geometry itself. More broadly, asymmetric coupled‐bend approaches in conventional waveguide systems have also shown that bend‐induced radiation loss can be reduced by engineering the modal environment of curved waveguides [40, 41]. However, these studies focused on auxiliary coupled curved waveguides and silicon nitride racetrack‐resonator configurations, leaving the problem of low‐loss routing in ultra‐compact silicon slot‐waveguide bends largely unexplored.

Here, we take an inverse design approach to ultra‐tight slot‐waveguide bends in which the inner and outer rail widths are allowed to evolve independently while maintaining a constant slot gap. Instead of enforcing a fixed slot‐confined profile throughout the entire curved section, the optimized solution develops rail‐width asymmetry near the bend center. This asymmetric geometry supports field redistribution toward the wider outer rail during propagation, followed by efficient coupling to the symmetric output slot mode. As a result, efficient guided transmission can be achieved under extreme curvature without requiring large bend radii or discrete strip–slot conversion between routing sections. The bend geometry is obtained through a particle swarm optimization framework combined with a modified Bezier curve parameterization [42, 43].

We validate this approach using three‐dimensional finite‐difference time‐domain (3D FDTD) simulations and experimental measurements on a 220‐nm‐thick silicon‐on‐insulator platform. The optimized 90° bend has a bending radius of 1.5 μm and a slot gap of 100 nm, while maintaining compatibility with symmetric input and output slot waveguides. Simulations predict quasi‐TE_0_ modal transmission exceeding 95% across the C‐band. Fabricated routing structures containing up to 80 cascaded ultra‐tight bends exhibit broadband transmission, with an effective excess loss of approximately 0.29 dB per 90° turn. These results demonstrate that inverse‐designed asymmetric mode evolution offers a practical design strategy for ultra‐compact slot‐waveguide routing in high‐density photonic integrated circuits.

## 2 Results

### 2.1 Geometry and Local Modal Characteristics of the Asymmetric Slot Waveguide Bend

Figure 1 summarizes the geometry and local modal characteristics of the proposed ultra‐compact slot waveguide bend. The device consists of two symmetric slot waveguide sections connected by an inverse‐designed asymmetric 90° bending region (Figure 1A). The asymmetric bend geometry was obtained using particle swarm optimization with a modified Bezier curve parameterization, as described in Methods. The optimized bend was designed for a bending radius of *R*_slot_ = 1.5 μm and a slot gap of *W*_g_ = 100 nm.

**FIGURE 1:**
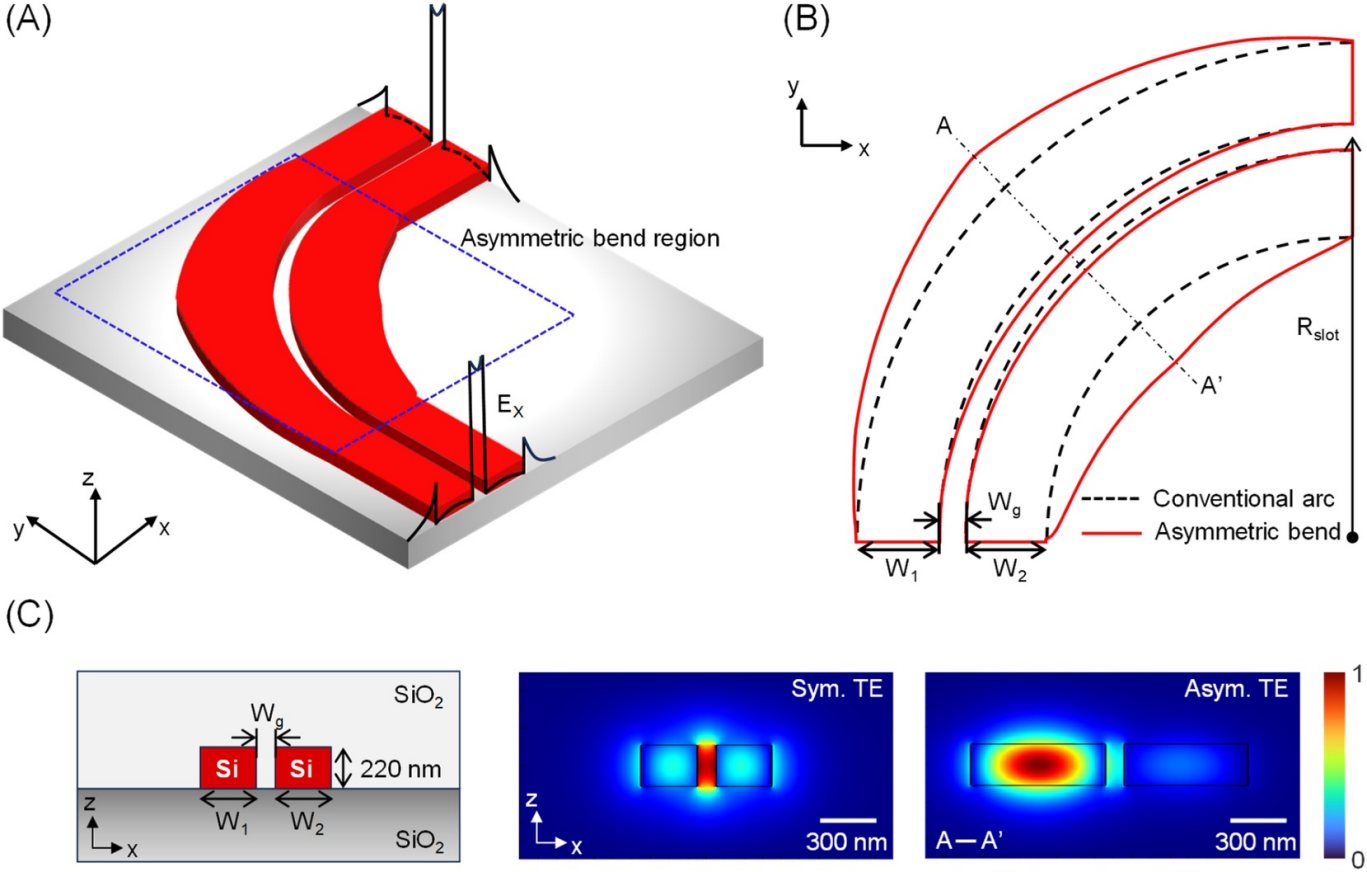
Geometry and local modal characteristics of the asymmetric slot waveguide bend (A) three‐dimensional schematic of the proposed 90° photonic slot waveguide bend. The dashed blue box denotes the asymmetric bend region connecting two symmetric slot waveguide sections. (B) Top‐view geometry comparison between a conventional arc‐shaped slot bend and the optimized asymmetric bend with *R*_slot_ = 1.5 μm and *W*_g_ = 100 nm. The A–A′ line indicates the bend‐center cross‐section used in (C). (C) Cross‐sectional geometry and local quasi‐TE_0_‐like eigenmode profiles at a wavelength of 1550 nm for the symmetric slot section and the asymmetric bend‐center section A–A′. The symmetric slot section has *W*_1_ = *W*_2_ = 300 nm, whereas the bend‐center section has *W*_1_ = 714 nm and *W*_2_ = 656 nm. The asymmetric geometry shifts the local quasi‐TE_0_‐like field profile toward the wider outer rail.

As shown in Figure 1B, the optimized geometry differs from a conventional arc‐shaped slot bend by introducing rail‐width variation along the curved section. While the two ends of the device remain compatible with symmetric slot waveguides, the bend‐center region forms an asymmetric cross‐section with unequal rail widths. This geometry provides a local modal environment that differs from that of a conventional symmetric slot waveguide.

To examine how this asymmetric geometry modifies the local modal profile, eigenmode calculations were performed for representative cross‐sections of the symmetric slot section and the bend‐center section A–A′. In the symmetric slot section, the quasi‐TE_0_ mode is concentrated in the low‐index slot gap, corresponding to the expected field distribution of a conventional slot waveguide (Figure 1C). In contrast, the asymmetric bend‐center section supports a local quasi‐TE_0_‐like eigenmode whose field profile is shifted toward the wider outer rail. This comparison shows that the optimized geometry induces local field reshaping at the bend center rather than preserving the same slot‐confined field profile throughout the entire structure.

It should be noted that the field profiles in Figure 1C are local eigenmode solutions of representative cross‐sections and do not by themselves describe the full propagation dynamics through the bend. They are used to illustrate the geometry‐induced tendency for field redistribution in the asymmetric bend region. The actual propagated field distribution is evaluated separately using three‐dimensional finite‐difference time‐domain simulations in Figure 2.

**FIGURE 2:**
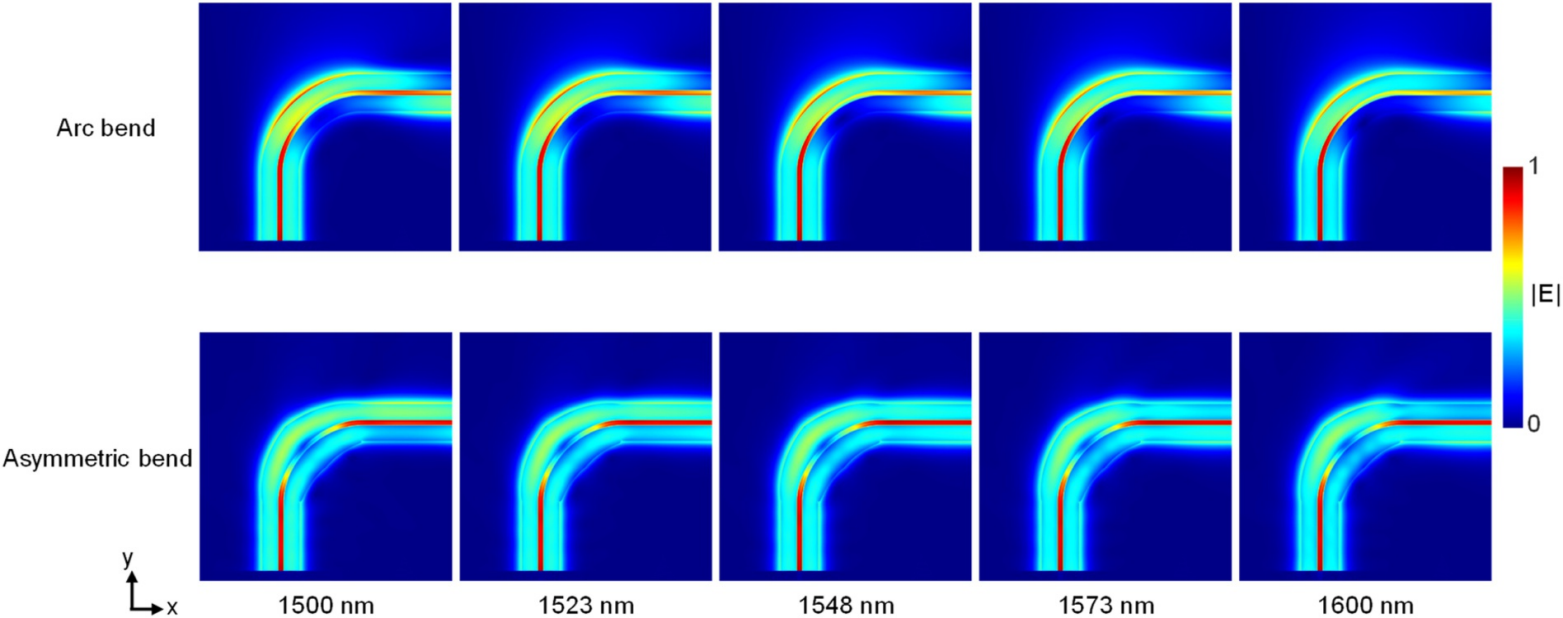
Wavelength‐dependent field distributions in conventional and asymmetric slot waveguide bends. Simulated electric‐field (E‐field) intensity distributions of conventional arc‐shaped slot waveguide bend (top row) and the optimized asymmetric bend (bottom row) at wavelengths of 1500, 1523, 1548, 1573, and 1600 nm. Both structures have the same bending radius (*R*_slot_ = 1.5 μm) and slot gap (*W*_g_ = 100 nm). The conventional arc bend shows outward field displacement and bend‐induced distortion, whereas the optimized asymmetric bend maintains a more continuous guided‐field distribution with optical intensity redistributed toward the outer rail.

Figure 2 compares the simulated electric‐field distributions of the conventional arc bend and the optimized asymmetric bend at multiple wavelengths over the 1500–1600 nm wavelength range. In the conventional bend, the field is displaced outward and exhibits pronounced distortion along the curved section. By contrast, the optimized asymmetric bend maintains a more continuous guided‐field distribution and shows optical intensity redistributed toward the outer rail over the wavelength range.

The physical role of the outer‐wide asymmetry can be understood in terms of local effective‐index compensation for curvature‐induced modal imbalance. In an ultra‐tight symmetric slot bend, the optical field is displaced toward the outer side of the bend, while the local symmetric slot geometry cannot fully accommodate this outward‐shifted field distribution, resulting in a mismatch with the local guided mode supported by the curved slot geometry. This mismatch leads to radiation leakage and inefficient projection onto the output quasi‐TE_0_ mode.

In the optimized geometry, the wider outer rail locally increases the effective refractive index on the outer side of the bend, thereby favoring redistribution of the outward‐shifted field toward the outer rail and reducing radiation leakage. The effective‐index analysis in Supporting Information S1 Figure S1 confirms that the asymmetric cross‐section modifies the local modal properties relative to the symmetric slot waveguide.

In addition, the cross‐sectional field distributions and transverse energy‐flow analysis in Supporting Information S1 Figure S2 show stronger asymmetric localization toward the wider outer rail and sign‐varying transverse energy redistribution, indicating energy‐flow reorganization rather than simple outward modal drift. As the inner and outer rail widths smoothly evolve back to the symmetric slot‐waveguide geometry toward the output, the redistributed field is gradually transferred back toward the slot region and efficiently recovered as the symmetric output quasi‐TE_0_ mode. This propagation‐field behavior is consistent with the local field‐reshaping tendency shown in Figure 1C, while the quantitative transmission performance is evaluated using mode‐expansion analysis in the following section.

### 2.2 Modal Transmission Characteristics of the Asymmetric Slot Waveguide Bend

To quantitatively assess how the asymmetric bend geometry affects guided‐mode transmission, we analyzed a single 90° slot waveguide bend using three‐dimensional finite‐difference time‐domain (3D FDTD) simulations. Figure 3 summarizes the wavelength‐dependent transmission characteristics obtained from mode‐expansion analysis for a bending radius of *R*_slot_ = 1.5 μm across the telecommunication C‐band.

**FIGURE 3:**
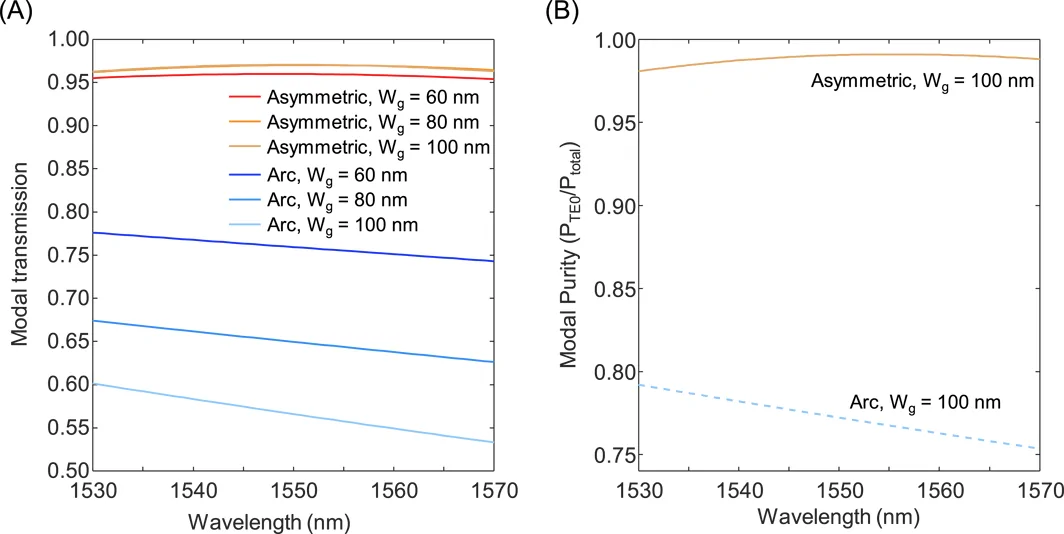
Single bend modal transmission and modal purity of the asymmetric slot waveguide bend. (A) Wavelength‐dependent quasi‐TE_0_ modal transmission obtained from mode‐expansion analysis for 90° slot waveguide bends with a bending radius of *R*_slot_ = 1.5 μm. Blue curves represent conventional arc‐shaped bends, and warm‐colored curves represent optimized asymmetric bends for slot gaps of 60, 80, and 100 nm. (B) Output modal purity, defined as $P_{\text{TE}0}/P_{\text{total}}$, for the conventional arc bend and the optimized bend with W_g_ = 100 nm. The optimized bend maintains both higher modal transmission and stronger preservation of the guided quasi‐TE_0_ mode across the C‐band.

Figure 3A shows the quasi‐TE_0_ modal transmission, which quantifies the optical power remaining in the guided quasi‐TE_0_ mode after the bend. Compared with conventional arc‐shaped bends, the optimized structures exhibit substantially improved transmission over the entire wavelength range for all considered slot gaps of 60, 80, and 100 nm. For the 100 nm slot‐gap case, the quasi‐TE_0_ modal transmission remains above 95% throughout the C‐band, confirming that the optimized geometry preserves efficient guided transport even under extreme curvature.

The modal transmission in Figure 3A represents the quasi‐TE_0_‐to‐quasi‐TE_0_ transmission through the bend, whereas the modal purity in Figure 3B is defined as the fraction of the transmitted output power carried by the quasi‐TE_0_ mode. Figure 3B shows that the optimized asymmetric bend maintains higher output modal purity than the conventional arc bend. This indicates that the transmitted field is not only larger in magnitude but also more strongly concentrated in the intended quasi‐TE_0_ mode after propagation through the ultra‐tight bend. Additional mode‐decomposition analysis is provided in Supporting Information S1 Figure S3, which shows that the transmitted field is dominated by the quasi‐TE_0_ mode and that TE‐to‐TM polarization conversion is negligible. These results indicate that the improved performance mainly arises from more efficient quasi‐TE_0_ guided‐mode transport rather than from polarization conversion or coupling to unintended modes.

Although the full optimized parameter sets are provided in Supporting Information S1 Tables S1 and S2, the representative *R*_slot_ = 1.5 μm and *W*_g_ = 100 nm design provides a useful reference for the optimized geometry. In this case, the bend‐center rail widths are *W*_1_ = 714 nm and *W*_2_ = 656 nm, corresponding to a wider outer rail. Re‐optimization at the same bend radius for different slot‐gap values consistently yielded asymmetric profiles with a wider outer rail while maintaining high quasi‐TE_0_ transmission. However, this tendency is not universal across all bend radii. At the tighter *R*_slot_ = 1.0 μm radius, the optimized midpoint rail widths became nearly balanced, with a slightly wider inner rail. These results indicate that the broader design principle is curvature‐dependent asymmetric modal redistribution rather than a single universal outer‐wide profile, with the detailed geometry determined by the specific curvature and slot‐gap conditions within the present Bezier‐parameterized design space.

In addition, we evaluated the fabrication tolerance of the optimized *R*_slot_ = 1.5 μm bend by perturbing the nominal geometry without re‐optimization. As shown in Supporting Information S1 Figure S4, the optimized bend preserves high quasi‐TE_0_ modal transmission under slot‐gap variations of ± 10 nm, uniform rail‐width variations of ± 10 nm applied to both the inner and outer rails, and device‐layer thickness variations of ± 5 nm. Even under these perturbations, the modal transmission remains above approximately 92% across the C‐band. These results indicate that the optimized asymmetric geometry retains reasonably high performance under moderate fabrication‐induced deviations, supporting its practical use in ultra‐compact silicon slot‐waveguide routing.

### 2.3 Experimental Demonstration of Cascaded Ultra‐Tight Slot Waveguide Bends

To experimentally validate the proposed ultra‐compact slot waveguide bend design, devices were fabricated on a silicon‐on‐insulator platform with a 220‐nm‐thick silicon device layer using a multi‐project wafer (MPW) process. Figure 4 shows optical microscope images of the fabricated devices corresponding to three representative routing configurations. Case 1 consists of a meander‐line geometry incorporating 20 ultra‐tight slot waveguide bends, whereas Cases 2 and 3 employ cascaded optimized bend structures with 40 and 80 turns, respectively. All devices share the same optimized slot‐bend profile with a bending radius of 1.5 μm, as highlighted in the inset scanning electron microscope image.

**FIGURE 4:**
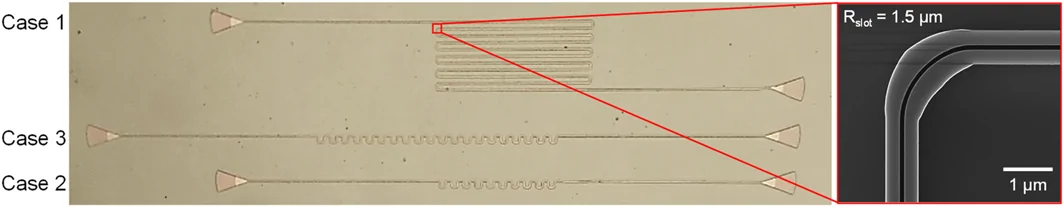
Optical microscope images of the three fabricated device cases. Case 1 consists of a 20‐turn meander‐line waveguide with a lateral spacing of 170 μm and a vertical pitch of 3 μm. Cases 2 and 3 employ optimized cascaded bend geometries with 40 and 80 turns, respectively, designed with a coupling length L_C_ = 3 μm. All devices share the same optimized bend profile with a bending radius of *R*_slot_ = 1.5 μm, as highlighted in the inset SEM image.

Optical transmission measurements were performed using a fiber‐coupled setup under controlled polarization to selectively excite the quasi‐TE_0_ mode. Light was coupled into and out of the chip via identical fully etched grating couplers. To isolate routing‐induced loss, the measured transmission spectra were normalized using an on‐chip reference structure consisting of identical grating couplers, a strip‐to‐slot mode converter pair, and a short straight slot waveguide section. Additional details of the normalization structure and the strip‐to‐slot converter are provided in Supporting Information S1 Figures S5 and S6, respectively.

Figure 5A shows the normalized transmission spectra of the three routing configurations over the telecommunication C‐band. Despite the large number of ultra‐tight bends, all measured devices exhibit clear broadband transmission. As expected, the normalized transmission decreases as the number of cascaded bends increases from 20 to 80, while the overall spectral response remains continuous across the measured wavelength range.

**FIGURE 5:**
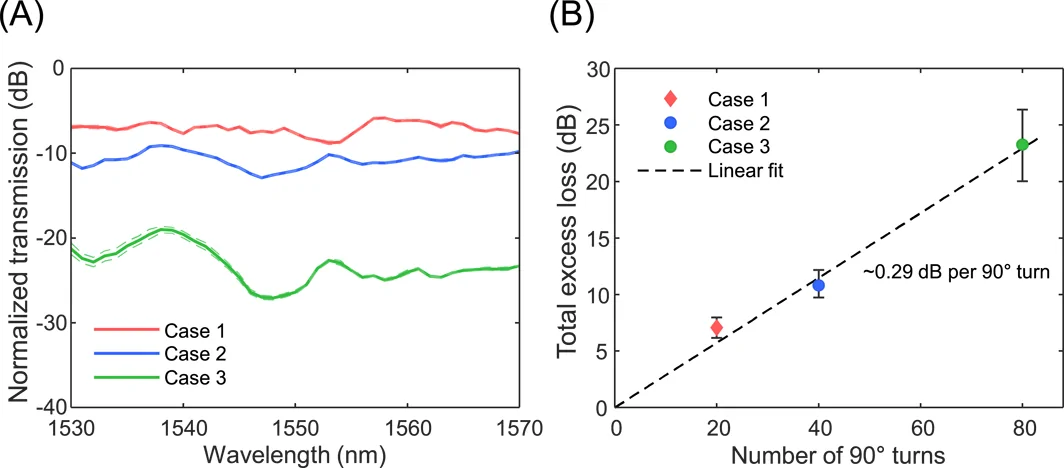
Normalized transmission and excess loss of cascaded asymmetric slot bend waveguides. (A) Wavelength‐dependent normalized transmission over the telecommunication C‐band (1530–1570 nm) for the three routing configurations shown in Figure 4: Case 1 (20‐turn meander‐line geometry), Case 2 (40 cascaded bends), and Case 3 (80 cascaded bends). Dashed curves indicate the ± 1σ variation obtained from three repeated measurements. All spectra were normalized using an on‐chip reference structure with identical input and output grating couplers. (B) Total excess loss extracted from the normalized transmission data in (A), plotted as a function of the number of 90° turns. Error bars indicate the wavelength‐dependent variation within the 1530–1570 nm band, represented by the 10th–90th percentile range. The dashed line indicates a linear fit used to extract the effective excess loss per 90° turn.

To quantify routing‐induced loss accumulation, Figure 5B plots the total excess loss as a function of the number of 90° turns. The cascaded‐bend configurations show an approximately linear increase in excess loss with increasing turn number. A linear fit gives an effective excess loss of approximately 0.29 dB per 90° turn. The error bars represent wavelength‐dependent variation within the 1530–1570 nm band, expressed as the 10th–90th percentile range.

For comparison, conventional arc‐shaped slot bends fabricated and measured under the same conditions exhibit severely reduced raw transmission, as shown in Supporting Information S1 Figure S7. This result is consistent with the lower simulated modal transmission of the conventional arc bend in Figure 3. Together, the simulation and experimental results confirm that the optimized asymmetric bend provides a practical ultra‐compact routing element for cascaded slot‐waveguide layouts.

## 3 Discussion and Conclusion

Tight bending remains a major limitation for slot‐waveguide routing because the slot‐confined mode is highly sensitive to curvature‐induced distortion. In conventional arc‐shaped slot bends, strong curvature can displace the field away from the slot region and reduce coupling to the desired output mode. This effect becomes especially severe at micrometer‐scale bending radii in high‐index‐contrast platforms such as silicon‐on‐insulator, where compact routing is desirable but bend‐induced modal distortion can be significant.

The inverse‐designed bend geometry addresses this limitation by using the independent rail‐width degrees of freedom available in the design space. Rather than enforcing an identical slot‐confined profile throughout the entire bend, the optimized geometry develops a gradual rail‐width asymmetry across the bending region, which becomes most pronounced near the bend center and shifts the local quasi‐TE_0_‐like field profile toward the wider outer rail. Three‐dimensional FDTD simulations show that the propagated field exhibits a similar asymmetric redistribution along the curved section, while mode‐expansion analysis confirms efficient coupling to the output quasi‐TE_0_ mode. This interpretation is supported by the combined evidence from local eigenmode analysis, propagation‐field distributions, and modal transmission calculations.

The numerical results show that the optimized asymmetric bend maintains high quasi‐TE_0_ modal transmission and output modal purity over the investigated wavelength range. For a bending radius of *R*_slot_ = 1.5 μm and a slot gap of *W*_g_ = 100 nm, the simulated quasi‐TE_0_ modal transmission exceeds 95%. Re‐optimized designs for different slot gaps also exhibit similar transmission characteristics, indicating that the asymmetric bend design is not restricted to a single slot‐gap condition. Additional simulations further show that the device remains tolerant to representative fabrication variations, including changes in slot gap, rail width, and device‐layer thickness.

Experimental measurements validate the compact routing capability of the proposed design. Routing structures incorporating up to 80 cascaded ultra‐tight bends exhibit clear broadband transmission across the C‐band after normalization to an on‐chip reference structure. The accumulated excess loss increases approximately linearly with the number of turns, corresponding to an effective excess loss of about 0.29 dB per 90° bend. Conventional arc‐shaped slot bends fabricated and measured under the same conditions show severely reduced transmission, consistent with their lower simulated modal transmission under the same bending radius and slot‐gap conditions.

Beyond compact routing, the asymmetric cross‐section may provide additional opportunities for local field engineering in slot‐waveguide platforms, as illustrated by the supporting analysis in Supporting Information S1 Figure S8. This aspect is not the primary focus of the present work, but it suggests that asymmetric slot geometries may be useful when compact routing and local field control are both required.

In summary, we experimentally demonstrate an ultra‐compact photonic slot waveguide bend based on an inverse‐designed asymmetric geometry. The proposed 90° bend operates at *R*_slot_ = 1.5 μm with a 100 nm slot gap and supports high simulated quasi‐TE_0_ modal transmission as well as broadband transmission in cascaded experimental routing structures. By combining inverse design, local modal analysis, 3D FDTD simulations, and experimental validation, this work establishes asymmetric mode evolution as a practical design strategy for ultra‐compact slot‐waveguide routing in integrated photonic platforms. This design concept may also be applicable to other integrated slot‐waveguide platforms, including silicon nitride (SiN) waveguides and hybrid slot geometries.

## 4 Methods

### 4.1 Inverse‐Designed Asymmetric Slot‐Bend Geometry and Optimization Framework

Among various waveguide bending geometries such as conventional circular, Bezier, Euler, and hybrid designs, a cubic Bezier curve was selected as the base geometry for parameterizing the slot‐waveguide bend. While a standard cubic Bezier curve is defined by four control points [43], in our design, the center trajectories of the inner and outer rails were defined using effective radii $R_{\text{eff}}$, corresponding to the center positions of each rail. For each rail, the Bezier control points were defined as (0,0), $(0,{\left(1-B\right)R}_{\text{eff}}$), $({BR}_{\text{eff}},R_{\text{eff}}$), and $(R_{\text{eff}},R_{\text{eff}}$), where B is a dimensionless shaping parameter controlling the curvature profile and varied within the range of 0.1–0.5.

To introduce asymmetric rail‐width modulation while maintaining a constant slot gap, four additional design parameters were used: the midpoint rail widths $W_{\text{mid},\text{out}}$ and $W_{\text{mid},\text{in}}$, and the width‐evolution coefficients $D_{1}$ and $D_{2}$. The midpoint rail widths were varied within the range of 0.1–1.0 μm, and $D_{1}$ and $D_{2}$ were varied within the range of 0.5–10. The input and output rail widths were fixed at $W_{1}=W_{2}=0.3\;\mu m$, which is a typical value for silicon slot waveguides supporting strong field confinement in the slot region. Along the normalized propagation coordinate i∈[0,1], the outer and inner rail widths were defined as

(1) $$W_{i,\text{out}}=W_{1}+\left(W_{\text{mid},\text{out}}-W_{1}\right){\left(1-2\left(\left|i-0.5\right|\right)\right)}^{D_{1}}$$

(2) $$W_{i,\text{in}}=W_{2}+\left(W_{\text{mid},\text{in}}-W_{2}\right){\left(1-2\left(\left|i-0.5\right|\right)\right)}^{D_{2}}$$

where $W_{i,\text{out}}$ and $W_{i,\text{in}}$ are the outer and inner rail widths, respectively, at normalized position i. The exponents $D_{1}$ and $D_{2}$ control how rapidly each rail width evolves from the symmetric input section to the asymmetric bend‐center section and back to the symmetric output section.

Particle swarm optimization (PSO) implemented in Ansys Lumerical was used to determine the optimal bend geometry. The optimization was performed using a swarm size of 30 particles and a maximum of 300 generations with a convergence tolerance of 1 × 10^−4^. The five geometric parameters were optimized simultaneously at a wavelength of 1550 nm. The figure of merit (FoM) was defined to minimize loss of the quasi‐TE_0_ mode while suppressing coupling into unwanted field components, including higher‐order or non‐guided modes, as defined in Equation (3).

(3) $$\text{FoM}=\left(1-\alpha \right)\left(1-\eta_{{\text{TE}}_{0}}\right)+\alpha \left(T-\eta_{{\text{TE}}_{0}}\right)$$

Here, $\eta_{{\text{TE}}_{0}}$ is defined as the power coupled to the desired output quasi‐TE_0_ mode normalized to the input power, whereas T represents the total transmitted power normalized to the input power. Therefore, $\eta_{{\text{TE}}_{0}}$ represents the quasi‐TE_0_ modal transmission through the bend rather than the output modal purity. The output modal purity is evaluated separately in Figure 3B as P_TE0_/P_total_, where P_TE0_ denotes the transmitted power carried by the output quasi‐TE_0_ mode and P_total_ denotes the sum of the transmitted powers carried by all output modes included in the mode‐expansion analysis. The weighting factor *α* = 0.4 was chosen as a practical compromise between high quasi‐TE_0_ modal transmission and suppression of nontarget transmitted power, while placing slightly greater emphasis on the desired‐mode transmission. A smaller *α* weakens the penalty on undesired transmitted components, whereas a larger *α* places greater emphasis on modal selectivity. Additional optimizations with different *α* values are provided in Supporting Information S1 Figure S9, showing that the high quasi‐TE_0_ transmission behavior is not unduly sensitive to moderate variations in *α*.

### 4.2 Electromagnetic Simulation and Modal Analysis

Numerical simulations were performed using a combination of eigenmode analysis and three‐dimensional finite‐difference time‐domain (3D FDTD) simulations implemented in Ansys Lumerical. Eigenmode calculations were first performed for representative cross‐sections of the symmetric slot waveguide and the asymmetric bend‐center section. These calculations were used to examine the local quasi‐TE_0_‐like modal profiles shown in Figure 1C. The local eigenmode analysis characterizes the modal environment of representative cross‐sections and is distinct from the full propagation simulations through the bend.

After obtaining the optimized asymmetric bend geometry through inverse design, full‐wave 3D FDTD simulations were carried out to analyze the field evolution and transmission characteristics of a single 90° slot waveguide bend. A quasi‐TE_0_ mode was launched from the symmetric input slot waveguide, and the transmitted field was analyzed at the symmetric output slot waveguide using mode‐expansion analysis. For the transmission simulations, a uniform mesh size of 10 nm was used in the bend region to resolve the 100 nm slot gap and the subwavelength rail‐width variations.

The quasi‐TE_0_ modal transmission was defined as the power coupled into the output quasi‐TE_0_ mode normalized to the input power. The output modal purity was defined as $P_{\text{TE}0}/P_{\text{total}}$, where $P_{\text{TE}0}$ is the output power carried by the quasi‐TE_0_ mode and $P_{\text{total}}$ is the total transmitted output power. Additional mode‐decomposition analysis was performed to evaluate coupling to the quasi‐TM_0_ mode and TE‐to‐TM polarization crosstalk. Supporting analyses, including rotated cross‐sectional field profiles, transverse energy‐flow distributions, and fabrication‐tolerance simulations, are provided in Supporting Information S1.

### 4.3 CMOS Compatible Fabrication

The devices were fabricated through a shared multi‐project wafer (MPW) run at Applied Nanotools (ANT) Inc., utilizing high‐resolution 100‐keV electron‐beam lithography. The patterns were defined on a standard silicon‐on‐insulator (SOI) wafer consisting of a 220‐nm‐thick silicon device layer, a 2 μm buried oxide layer, and a 725 μm silicon substrate. Prior to exposure, the wafer surface was cleaned and coated with an electron beam sensitive resist. The designed layouts were subsequently written directly into the resist layer and transferred into the silicon device layer through anisotropic ICP‐RIE etching, which proceeded until the buried oxide was reached, thereby yielding a fully etched device layer. In this fabrication process, the minimum feature size was 60 nm, and the minimum feature spacing was 70 nm when operating under < 50% duty cycle exposure conditions. Following etching, a 2.2 μm‐thick SiO_2_ overcladding was deposited using a plasma enhanced chemical vapor deposition (PECVD) to complete device passivation.

### 4.4 Experimental Setup

Optical characterization was carried out using a fiber‐coupled optical measurement setup. A continuous wave tunable laser source (Agilent 81949A) delivered an input power of 6 dBm through a single mode fiber. The input polarization state was adjusted using a polarization controller to selectively excite the quasi‐TE_0_ mode. Light was coupled into the chip through a fully etched grating coupler under an 8° incidence angle. The transmitted signal was collected by an output grating coupler and detected using an InGaAs power meter (Agilent 81635A). Wavelength‐dependent transmission spectra were recorded by sweeping the laser wavelength. The overall insertion loss, including the input/output grating couplers and on‐chip propagation, was approximately 22 dB at 1550 nm; additional details are provided in Supporting Information S1 Figure S5.

## Author Contributions

**Ji‐Hwan Park:** conceptualization, methodology, software, data curation, investigation, validation, formal analysis, visualization, writing – original draft, writing – review and editing. **Jae‐Yong Kim:** software. **Seokjin Hong:** validation, writing – review and editing. **Berkay Neseli:** resources. **Hyo‐Hoon Park:** funding acquisition. **Hamza Kurt:** supervision, writing – review and editing, funding acquisition, validation.

## Conflicts of Interest

The authors declare no conflicts of interest.

## Supporting information


Supporting Information S1

## Data Availability

The data that support the findings of this study are available from the corresponding author upon reasonable request.
