# Supplementary material for: Low‐Loss Routing of Strongly Confined Light in Ultra‐Compact Silicon Slot‐Waveguide Bends
Source: Nanophotonics. 2026 Aug 20;15(16):e70260. doi: 10.1002/nap2.70260 (PMC13672125; doi:10.1002/nap2.70260)
Supplement: Supplementary file 1 [file NAP2-15-e70260-s001.docx]

Supplementary Materials for

**Low-Loss Routing of Strongly Confined Light in Ultra-Compact Silicon Slot-Waveguide Bends**

Ji-Hwan Park^1,2^, Jae-Yong Kim^1^, Seokjin Hong^1^, Berkay Neseli^1^, Hyo-Hoon Park^1^ and Hamza Kurt^1^**.*

*Corresponding author. Email: hamzakurt@kaist.ac.kr

^1^ School of Electrical Engineering, Korea Advanced Institute of Science and Technology, Daejeon 34141, Korea

^2^ Command, Control, Communication, Computer, Intelligence, Surveillance and Reconnaissance (C4ISR) Systems Center, Defense Agency for Technology and Quality (DTaQ), Daegu 42037, Korea

| **(a)** | **(b)** |
| --- | --- |

Fig. S1. Modal properties of the asymmetric slot cross-section and radius-dependent optimization behavior.

**(a)** Simulated effective refractive indices of the quasi-TE_0_ and quasi-TM_0_ modes for the symmetric and asymmetric slot-waveguide cross-sections over the wavelength range of 1500–1600 nm.

**(b)** Evolution of the figure of merit (FoM) during particle swarm optimization for representative bend radii of R_slot_ = 1, 1.5, and 2 µm.

**Supplementary text**

Figure S1 supports the design rationale presented in the main text. Panel (a) shows that the asymmetric cross-section modifies the local modal properties relative to the symmetric slot waveguide, consistent with the field redistribution observed near the bend center. Panel (b) shows that, among the examined radii, the R_slot_ = 1.5 µm case provides the most favorable optimization outcome under the same design framework. These results support the selection of R_slot_ = 1.5 µm as the reference geometry used throughout the main text.

| **Fixed value** | | | **Optimization Results** | | | | | |
| --- | --- | --- | --- | --- | --- | --- | --- | --- |
| **R_slot_ (µm)** | **In/out rail width (nm)** | **Slot gap**  **(nm)** | **W_out,mid (µm)** | **W_in,mid (µm)** | **B** | **D_1_** | **D_2_** | **FoM** |
| **1.0** | **300** | **100** | **0.6315** | **0.647502** | **0.5** | **0.743717** | **0.5** | **0.1304** |
| **1.5** | **300** | **100** | **0.714291** | **0.656103** | **0.499423** | **0.977242** | **0.5** | **0.0328** |
| **2.0** | **300** | **100** | **0.719818** | **0.696675** | **0.5** | **1.37514** | **0.989263** | **0.0940** |

Table S1. Radius-dependent optimized geometric parameters of inverse-designed slot waveguide bends.

**Supplementary text**

Table S1 summarizes the optimized geometric parameters obtained for representative bend radii of 1, 1.5, and 2 µm under the same design framework. Although the same initial rail width and slot gap were used in all cases, the optimized midpoint rail widths and modulation parameters vary with bend radius. These results show that the final asymmetric bend geometry depends systematically on the selected radius, providing additional flexibility for controlling modal redistribution in ultra-compact slot waveguide bends.

| **(a)** | **(b)** |
| --- | --- |
| **(c)** | **(d)** |

Fig. S2. Cross-sectional field distributions and transverse energy flow analysis in conventional arc and asymmetric slot waveguide bend.

(a) Normalized $\mid E\mid^{2}$cross-sectional map of a conventional arc-shaped slot waveguide bend obtained along the rotated $u$-axis.

(b) Signed lateral Poynting component $S_{u}$ for the conventional arc bend.

(c) Normalized $\mid E\mid^{2}$ cross-section of the optimized asymmetric bend extract under the same rotated slicing condition.

(d) Signed $S_{u}$ distribution for the optimized bend. Compared with the conventional arc bend, the optimized asymmetric structure exhibits stronger asymmetric field localization and more pronounced sign-varying transverse energy redistribution.

**Supplementary text**

To further examine the physical difference between the conventional and optimized bends, cross-sectional field profiles and transverse energy flow were analyzed using a rotated local coordinate system aligned with the outward direction of the bend. As shown in Fig. S2(a, c), the conventional arc bend retains a more slot-like profile with outward-shifted intensity, whereas the optimized bend exhibits stronger asymmetric localization toward the wider outer rail. The corresponding $S_{u}$ distributions in Fig. S2(b, d) show that the arc bend is dominated by outward-directed transverse energy flow, while the optimized bend exhibits a pronounced sign-varying redistribution within the cross-section. These results suggest that the optimized bend involves asymmetric field redistribution and transverse energy-flow reorganization, rather than simple outward modal drift. Because the cross-sections are extracted along a rotated axis, the apparent rail widths in the plots may appear smaller than the actual physical widths. The signed Poynting component projected along the rotated $u$-axis is evaluated as

$$\boldsymbol{S}_{\boldsymbol{u}}=\frac{1}{2}\mathrm{Re}\{(\mathbf{E}\times\mathbf{H}^{*})\cdot\hat{\mathbf{u}}\}.$$

The quantity $S_{u}$ is normalized by its maximum magnitude to enable direct comparison across different geometries.

| **Fixed value** | | | **Optimization Results** | | | | | |
| --- | --- | --- | --- | --- | --- | --- | --- | --- |
| **R_slot_ (µm)** | **In/out rail width (nm)** | **Slot gap**  **(nm)** | **W_out,mid (µm)** | **W_in,mid (µm)** | **B** | **D_1_** | **D_2_** | **FoM** |
| **1.5** | **300** | **60** | **0.645601** | **0.612117** | **0.5** | **0.5** | **0.5** | **0.0322** |
| **1.5** | **300** | **80** | **0.706453** | **0.637249** | **0.5** | **0.817863** | **0.5** | **0.0298** |
| **1.5** | **300** | **100** | **0.714291** | **0.656103** | **0.499423** | **0.977242** | **0.5** | **0.0328** |

Table S2. Re-optimized geometric parameters of inverse-designed slot waveguide bends for different slot-gap values.

**Supplementary text**

Table S2 summarizes the optimized geometric parameters obtained for representative slot-gap values of 60, 80, and 100 nm under the same design framework. Although the optimized midpoint rail widths and modulation parameters vary with slot gap, high transmission performance is maintained across all cases. These results show that the proposed bend design can be systematically adapted to different slot-gap conditions.

| **(a)** | **(b)** |
| --- | --- |

Fig. S3. Modal decomposition and polarization crosstalk analysis of slot waveguide bends.

(a) Modal transmission projected onto the quasi-TE₀ and quasi-TM₀ modes for quasi-TE₀ excitation, obtained from mode-expansion analysis at the output slot waveguide for the optimized bend.

(b) Polarization crosstalk defined as

$$\mathrm{XT}_{\mathrm{TE}\to\mathrm{TM}}=10\log_{10}\left( \frac{P_{\mathrm{TM}0}}{P_{\mathrm{TE}0}} \right),$$

where $P_{\mathrm{TE}0}$and $P_{\mathrm{TM}0}$ are the output powers projected onto the quasi-TE₀ and quasi-TM₀ modes, respectively. The results demonstrate negligible coupling from the quasi-TE₀ mode to the quasi-TM₀ mode across the operating wavelength range

**Supplementary text**

To further quantify the modal behavior of the bend structures, we analyze the transmitted field using mode-expansion analysis at the output monitor. The output field is decomposed into contributions from the quasi-TE₀ and quasi-TM₀ modes, enabling direct evaluation of modal transmission, modal purity, and polarization crosstalk.

As shown in Fig. S3(a), the optimized bend exhibits a transmitted field that is strongly dominated by the quasi-TE₀ mode across the entire wavelength range, while the quasi-TM₀ component is negligible. This indicates that polarization conversion is effectively suppressed despite the strong structural asymmetry and ultra-tight curvature.

Figure S3(b) further quantifies the polarization crosstalk, showing that TE-to-TM conversion is negligible. This behavior is consistent with the strong birefringence of the slot waveguide platform, which results in a large effective-index mismatch between the quasi-TE₀ and quasi-TM₀ modes and therefore suppresses inter-polarization coupling.

These results confirm that the transmitted field is dominated by the quasi-TE₀ mode, while TE-to-TM polarization conversion remains negligible across the operating wavelength range. Therefore, the residual loss is mainly associated with radiation and mode-mismatch losses rather than polarization conversion.

Fig. S4. Fabrication tolerance analysis of the optimized slot waveguide bend (R_slot_ = 1.5 µm).

(a) Transmission spectra under slot-gap variations of ±10 nm applied to the nominally optimized structure without re-optimization.

(b) Transmission spectra under global rail-width variations of ±10 nm applied uniformly to all waveguide sections, including the optimized bend region, without re-optimization.

(c) Transmission spectra under device-layer thickness (etch-depth) variations of ±5 nm, also evaluated without re-optimization.

In all cases, the nominally optimized structure at R_slot_ = 1.5 µm was directly perturbed to emulate fabrication-induced deviations while maintaining identical simulation and analysis conditions. The results show that the proposed design preserves high quasi-TE_0_ modal transmission under realistic geometric perturbations.

**Supplementary text**

To evaluate fabrication tolerance, we performed additional 3D FDTD simulations by introducing representative geometric perturbations to the nominally optimized structure without re-optimization, as summarized in Fig. S4.

First, the slot gap was varied by ±10 nm relative to the nominal design. As shown in Fig. S4(a), the transmission spectra exhibit only minor deviations from the nominal case, indicating that the optimized bend remains robust against moderate slot-gap perturbations arising from fabrication errors.

Second, a global rail-width bias of ±10 nm was applied uniformly to all waveguide sections, including both the input/output straight regions and the optimized bend region. This perturbation models typical critical-dimension variations introduced during lithographic fabrication. As shown in Fig. S4(b), the transmission remains only weakly affected, confirming limited sensitivity to moderate rail-width deviations.

Finally, the device-layer thickness was varied by ±5 nm to account for etch-depth fluctuations in the vertical fabrication process. As shown in Fig. S4(c), the transmission remains largely unchanged, demonstrating that the device is also tolerant to moderate variations in vertical confinement.

These results confirm that the proposed bend design is robust against realistic fabrication-induced perturbations. Although the slot gap remains the most influential parameter governing modal confinement, moderate variations in slot gap, rail width and etch depth produce only limited degradation in transmission, indicating that the optimized asymmetric geometry provides practical tolerance to fabrication errors.

| **(a)** | |
| --- | --- |
| **(b)** | **(c)** |

Fig. S5. On-chip normalization structure and validation measurement.

(a) Layout of the validation and normalization structures fabricated in the same chip. Top: a straight strip-waveguide validation structure (600 µm) connected to identical input/output fully etched grating couplers designed using the foundry process design kit and optimized for transverse-electric (TE) coupling under an 8° fiber incidence angle. Bottom: the normalization structure with the same total length, incorporating a strip-to-slot mode-converter (S2S converter) pair and a 9 µm straight slot waveguide section between the converters. An SEM image of the S2S converter is shown in the inset.

(b) Raw detected power spectra (raw transmission) of the strip validation and normalization structures measured using the same fiber-coupled setup.

(c) Difference spectrum obtained by subtracting the strip validation trace from the normalization trace, shown as ΔP (Normalization − Strip) in dB, confirming negligible wavelength-dependent deviation introduced by the normalization structure.

**Supplementary text**

All structures employ the same input/output fully etched grating couplers designed using the foundry process design kit (PDK), optimized for coupling to the transverse-electric polarization under an 8° fiber incidence angle. The spectra were recorded by sweeping the tunable laser wavelength with a step size of 1 nm over 1530–1570 nm. The normalization structure exhibits a raw detected-power spectrum that closely follows the spectral trend of the straight strip validation structure. To quantify potential wavelength-dependent deviations introduced by the strip-to-slot (S2S) converter pair, a difference spectrum was calculated as ΔP = P_norm − P_strip (dB). The resulting ΔP remains within the measurement uncertainty across the C-band, indicating that the S2S converter pair does not introduce significant wavelength-dependent artifacts within the resolution of the experimental setup.

| **(a)** | **(b)** |
| --- | --- |
| **(c)** | |

Fig. S6. Design and characterization of the strip-to-slot (S2S) mode converter.

(a) FoM convergence obtained during the inverse-design optimization process used to generate the S2S converter geometry.

(b) Simulated electric-field amplitude distribution at 1550 nm, illustrating mode transition from strip to slot waveguide.

(c) Forward and backward fundamental-mode net transmission of the inverse-designed strip-to-slot (S2S) mode converter simulated over the 1500–1600 nm wavelength range. Blue and red curves correspond to forward excitation (strip→slot) and backward excitation (slot→strip), respectively. The close agreement between the two traces confirms reciprocal operation of the non-adiabatic ultra-compact converter used in normalization measurements.

**Supplementary text**

The strip-to-slot (S2S) mode converter was optimized primarily to ensure consistent normalization and mode interfacing during experimental measurements and is not the focus of this work. The optimization was performed using the particle swarm optimization (PSO) algorithm implemented in Ansys Lumerical, with the figure of merit defined as $\mathrm{FoM}=-10\log_{10}(T_{\mathrm{net}})$, where $T_{\mathrm{net}}$ denotes the fundamental mode net transmission.

The optimized converter geometry, shown in Fig. S6(b), maintains the same slot gap, rail widths, and strip-waveguide dimensions as the main devices, with a compact transition length of 1 µm. Figure S6(c) presents the simulated forward (strip→slot) and backward (slot→strip) fundamental-mode net transmission obtained from 2.5D variational FDTD simulations over the 1500–1600 nm wavelength range. The close agreement between forward and backward responses confirms reciprocal broadband operation of the converter, supporting its suitability for use in the normalization scheme.

Fig. S7. Experimental limitation of conventional slot waveguide bends due to cumulative loss.

(a) Measured raw detected-power spectrum of a conventional 20-turn meander-line slot waveguide bend within the wavelength range of 1530–1570 nm, showing severely reduced signal levels with no clearly resolved spectral features.

(b) Estimated accumulated bend loss for 20 turns, derived from the simulated single-turn transmission of the conventional arc-shaped bend.

For the conventional arc-shaped bend, only the 20-turn meander-line structure was experimentally characterized, because further cascading would reduce the transmitted signal toward the measurement noise floor. The reduced signal level is primarily attributed to the large bend-induced radiation loss of conventional arc geometry, as supported by the single-bend simulation results.

**Supplementary text**

To further clarify the experimental observability of the conventional slot waveguide bend, we analyzed its cumulative transmission behavior in Fig. S7. For the conventional arc-shaped bend, only a 20-turn meander-line structure was experimentally characterized. Within the wavelength range of 1530–1570 nm, the measured raw detected signal remains severely reduced and does not exhibit clearly resolved spectral features.

This behavior is consistent with the intrinsically large bend-induced loss of conventional arc geometry. As shown in Fig. 3, the simulated transmission of a single conventional arc-shaped bend is already significantly reduced due to strong curvature-induced radiation. Because numerical simulations represent idealized structures without fabrication imperfections or additional scattering mechanisms, the experimentally observed transmission is expected to be even lower.

To qualitatively illustrate this cumulative-loss behavior, the accumulated bend loss for 20 turns was estimated from the simulated single-turn transmission. This estimation reflects the monotonic accumulation of bend-induced loss and is not intended to provide quantitative agreement with the measured raw power.

These results indicate that the severely reduced signal level in the conventional structure is primarily caused by intrinsic bend-induced radiation loss, which limits the experimental observability of cascaded conventional slot bends.

| **(a)** | **(b)** | **(c)** |
| --- | --- | --- |

Fig. S8. Effective index and analyte confinement of asymmetric slot waveguides.

(a) Cross-sectional schematic of the asymmetric slot waveguide defining the analyte thickness $t_{a}$, slot gap of 100 nm, and rail widths $W_{1}$and $W_{2}$. The analyte layer is placed on the top surface of the wider rail and is surrounded by aqueous solution.

(b) Simulated effective refractive index of the quasi-TM₀ mode as a function of $t_{a}$at a wavelength of 1550 nm for three $\left( W_{1}, W_{2} \right)$ combinations.

(c) Corresponding analyte confinement factor, $\Gamma_{a}$, of the quasi-TM₀ mode as a function of $t_{a}$, showing enhanced surface field localization for asymmetric rail-width configurations.

**Supplementary text**

Figure S8 illustrates an additional implication of the asymmetric slot-waveguide cross-section that emerges in the optimized bend geometry. Although this analysis is not central to the routing performance discussed in the main text, it provides further insight into how rail-width asymmetry modifies local field confinement near the waveguide surface. In this analysis, the quasi-TM₀ mode was used as a probe mode because its stronger vertical electric-field component provides a clearer indication of surface-localized field redistribution induced by the asymmetric slot geometry.

The effective refractive index shown in Fig. S8(b) varies only modestly with analyte thickness, indicating that it is relatively insensitive to localized surface perturbations. To quantify the overlap more directly, the analyte confinement factor $\Gamma_{a}$ was defined as the fraction of optical energy residing within the analyte region:

$$\begin{matrix} & \Gamma_{a}=\frac{\iint_{\mathrm{analyte}} \mid E(x,z)\mid^{2}\text{ }dx\text{ }dz}{\iint_{\mathrm{total}} \mid E(x,z)\mid^{2}\text{ }dx\text{ }dz} & & \text{(S1)} \end{matrix}$$

Here, $E(x,z)$ denotes the electric-field distribution of the quasi-TM₀ mode. As shown in Fig. S8(c), the asymmetric configurations exhibit enhanced analyte confinement compared with more symmetric cases, indicating broader opportunities for engineering light–matter interaction in addition to compact routing.

Fig. S9. Sensitivity of the optimized slot-bend performance to the weighting factor α in the figure of merit. Quasi-TE_0_ modal transmission spectra are compared for independently optimized slot-waveguide bends with R_slot_ = 1.5 µm, obtained using α = 0.2, 0.4, and 0.6. The three optimized designs exhibit similar broadband transmission over the investigated wavelength range.

**Supplementary text**

To examine the sensitivity of the optimization outcome to the weighting factor α in the figure of merit, additional independent optimizations were performed for α = 0.2 and α = 0.6 and compared with the original α = 0.4 optimization. The same Bezier-parameterized rail-width design space and optimization procedure were used in all cases. As shown in Fig. S9, the resulting quasi-TE₀ modal transmission spectra exhibit only minor differences over the investigated wavelength range. This indicates that the high-transmission behavior of the optimized slot bend is not unduly sensitive to moderate variations of α. The result supports the interpretation that the main design principle is controlled asymmetric modal redistribution and output-mode recovery, rather than a solution that depends critically on a single weighting value.

REFERENCES

[S1] Y. Liu, M. Kong, and Y. Jiang, “Transverse magnetic modes in planar slot waveguides,” J. Opt. Soc. Am. B 32, 2052–2060 (2015).

[S2] C. G. Valdez, S. A. Bongarz, A. R. Kroo, A. J. Miller, M. J. F. Digonnet, D. A. B. Miller, and O. Solgaard, “Three-wave interaction grating coupler with the sub-decibel insertion loss at normal incidence,” Optica 13, 180–187 (2026).
